# Tribute to Prof. Geoffrey Burnstock: his contribution to acupuncture

**DOI:** 10.1007/s11302-020-09729-8

**Published:** 2020-10-09

**Authors:** Yong Tang, Peter Illes

**Affiliations:** 1 International Collaborative Center on Big Science Plan for Purine Signalling, Chengdu University of Traditional Chinese Medicine, Chengdu, 610075 China; 2 School of Acupuncture and Tuina, Chengdu University of Traditional Chinese Medicine, Chengdu, 610075 China; 3 Key Laboratory of Sichuan Province for Acupuncture & Chronobiology, Chengdu, 610075 China; 4 Rudolf-Boehm-Institut für Pharmakologie und Toxikologie, Universität Leipzig, 04107 Leipzig, Germany

**Keywords:** Tribute, Prof. Geoffrey Burnstock, Purinergic signalling

As the creator of purinergic signalling [1], Prof. Geoffrey Burnstock (1929–2020) [2] also made important contributions to the science of acupuncture (AP). AP family procedures include mechanical needling, electroacupuncture, moxibustion, cupping, etc. [3]. His insightful hypothesis on the natural scientific basis of AP in 2009 [4] led to an explosion of research worldwide that aimed to explaining how purinergic signalling contributes to AP. The development of our understanding on AP-induced analgesia has been elaborately documented in the past [5–7]; hence, in this article, we summarize Geoff’s distinguished contribution to, and profound impact on, AP and also disclose some personal accounts of his impact on our personal careers in specific, and on the Chinese AP community on a larger scale.

## Novel hypothesis on the purinergic mode of action of acupuncture

**Fig. 1:**
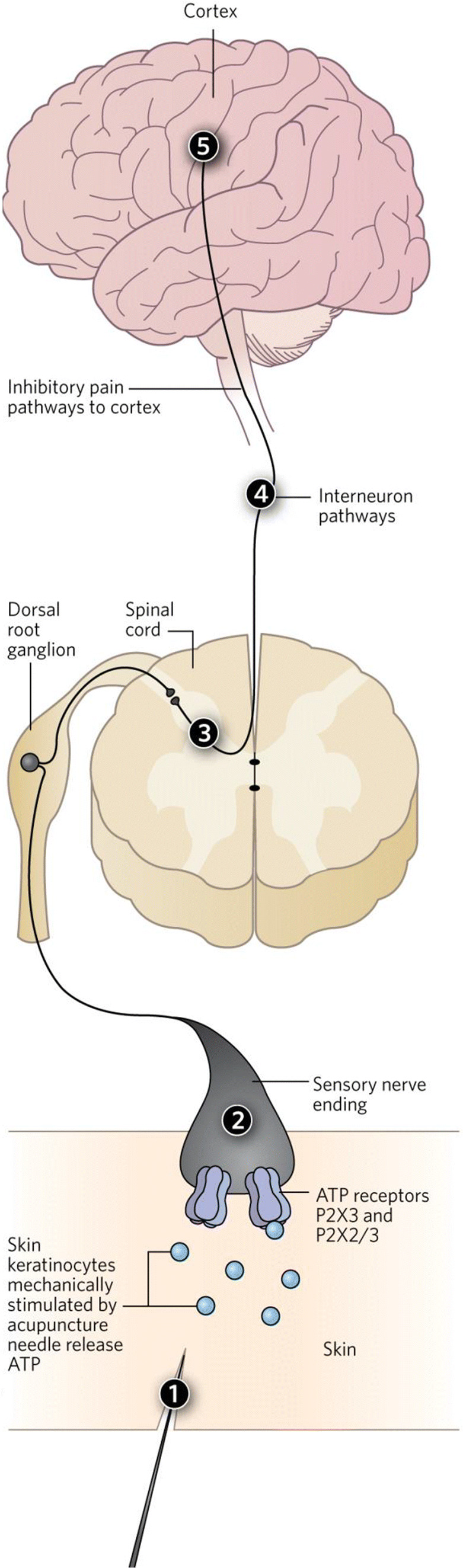
Purinergic hypothesis on acupuncture mechanism. For explanation, see text

AP family procedures, originally developed in China, are widely used in over 183 countries and regions all over the world. In 1980, 43 kinds of diseases were recommended by the WHO to be treated by AP, and this number was increased to 64 in 1996. In 1997, an NIH consensus was reached and communicated to the public, stating that AP verifiably works on nausea, vomiting, pain, and other conditions, based on evidence provided by clinical trials and the endorphin theory of its mode of action [8, 9]. However, the endorphin hypothesis still failed to completely explain how AP acts, and therefore, it was a major development when in 2009 Geoff published his hypothesis [4], later updated in 2011 [10] and 2014 [11]. He proposed that insertion and twisting of the needles employed in AP mechanically deform the skin, causing the local release of ATP by keratinocytes (1) (Fig. 1). The ATP binds to and activates P2X3 and P2X2/3 receptors located on sensory nerve endings in the skin (2) to initiate action potentials. The signal is then relayed via the dorsal root ganglia to the spinal cord (3) and subsequently through interneuronal pathways (4) to the brain stem (5), which contains motor neurons that control the activity of the gut, lungs, heart, arteries, and reproductive organs—all major targets for AP. Signals also travel to the pain centers of the cortex, delivering a message to inhibit pain (6) [11].

Subsequently, AP research in the field of purinergic signalling started to flourish and a large set of data has been accumulated on changes in purine (ATP, adenosine) concentrations, both locally and in the CNS, induced by manual acupuncture, electroacupuncture, or moxibustion [12–17]. The participation of purinergic receptors (P1: A1, A2a, A2b, A3) [18–34], (P2: P2X2, P2X3, P2X7, P2Y1, P2Y13) [35–52], and neuronal pathways bearing these receptors [53, 54] has been confirmed in different AP-sensitive conditions (pain, inflammation, myocardial ischemia, obesity, cerebral ischemia). The functionalized acupuncture needle as a SERS (surface-enhanced Raman spectroscopy)-active platform was developed for rapid and sensitive determination of ATP concentration [55], and led further support to the purinergic basis of AP.

It is worth pointing out that AP science has enormously benefited from two findings confirming purinergic mechanisms as participating in AP. The first is the evidence that local adenosine and adenosine A1 receptors mediate the anti-nociceptive effect of acupuncture in mice, as shown by Maiken Nedergaard and her group, which indicated that the needling site at an AP point is crucial to explain the mode of action of AP analgesia. The second was that the injection of prostatic acid phosphatase (PAP) to an AP point slowed down the degradation of adenosine and caused strong analgesia in mice, a procedure named “PAPupuncture” by Mark Zylka in 2012 [56]. Taken together, the purinergic hypothesis of AP mechanism has opened new vistas to understand what happens at AP points and is expected to uncover more valuable details in future. We still do not know which layer (skin, muscles, vessels, nerve endings, etc.), which cell types (keratinocytes, melanocytes, Merkel cells, Langerhans cells, fibroblasts, myocytes, vascular endothelial cells, etc.) at the AP point are responsible for AP effects. The release of purines in relation to the 14 main meridians of the human body, assumed to be tightly connected with AP according to Traditional Chinese Medicine (TCM), is also unknown. There are many further open questions: (1) Which of the purine degrading enzymes (CD39, CD73) have the largest impact on AP?; (2) Which of the P2 receptors (P2X1, P2X4, P2X5, P2X6, P2X7, P2Y2, P2Y4, P2Y6, P2Y11, P2Y12, P2Y13, P2Y14) are most important for AP?; and (3) Do P2X/Y receptors interact with other transmitter receptors in constituting AP effects? Especially the translational research from bench to bedside should be considered for AP research in the future.

## Important ideas to promote our knowledge on the natural scientific basis of acupuncture: Yong Tang (Chengdu, China)

Geoff delivered three inspiring lectures on purinergic signalling and AP in the course of his life, two in Chengdu and one in Leipzig. In the winter of 2011, based on the recommendation of Geoff, Peter Illes (Leipzig University, Germany) and Yong Tang (Chengdu University of TCM, China) worked together to write a proposal to the Sino-German Center for the Promotion of Science and obtained the funding for their common project in the spring of 2012.

**Fig. 2:**
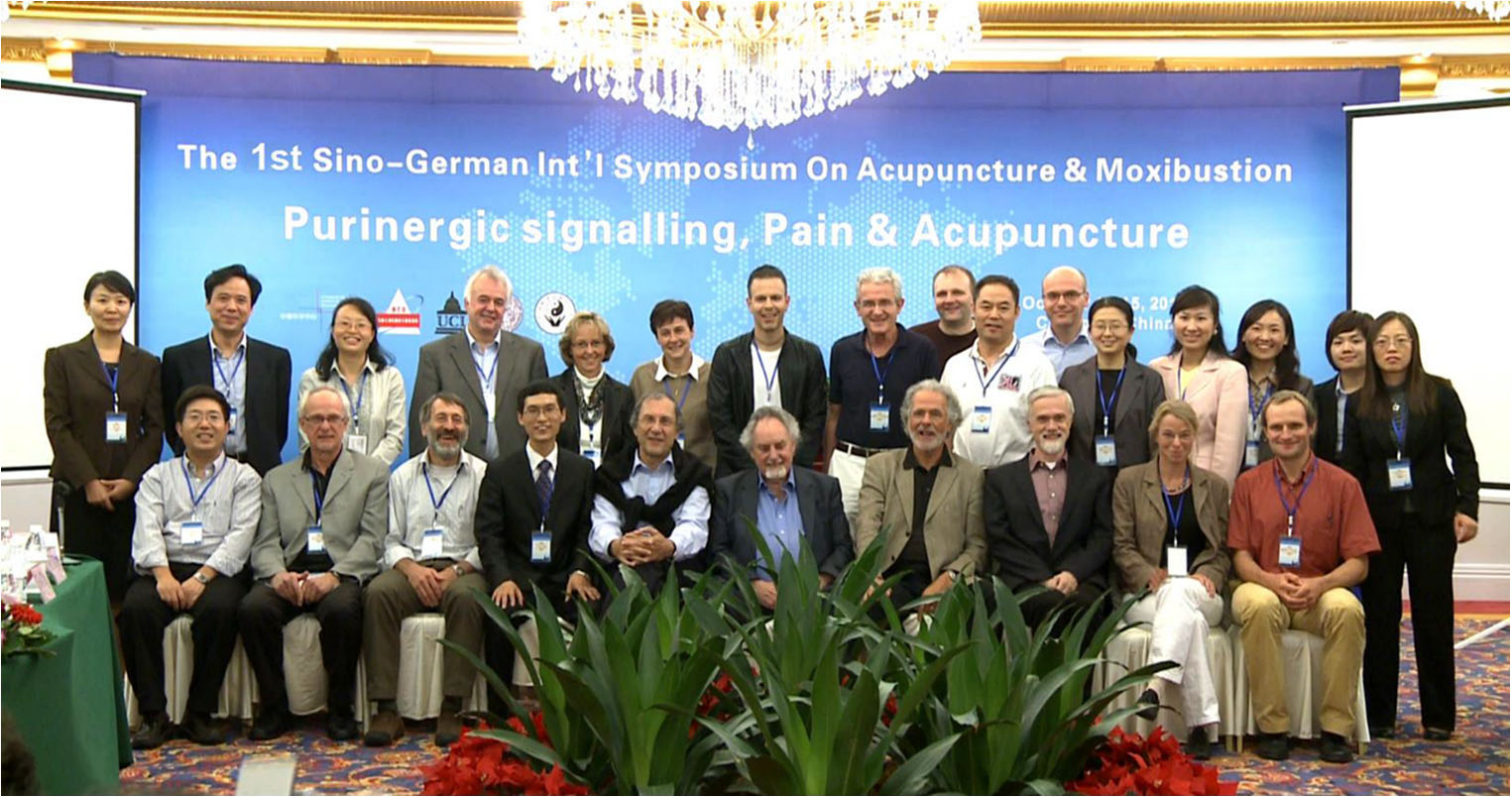
Geoffrey Burnstock, Peter Illes, and Yong Tang at the 1st Sino-German Symposium on Purinergic Signalling, Pain and Acupuncture in Chengdu, 2012. Reproduced with permission from [57]

Financed by this project, the three co-chairs (Geoff, Peter, and Yong) (Fig. 2), organized the “1st Sino-German Symposium on Purinergic Signalling, Pain and Acupuncture” in Chengdu between October 10 and 15, 2012. Geoff, at the age of 83, flew to China with his wife Nomi at the official invitation of the Chengdu University of TCM and opened this meeting with a 1-h introductory lecture. His excellent talk, focusing on the AP purinergic hypothesis, attracted a number of senior and junior scientists and students to the auditorium. During the conference, Geoff also met a number of AP researchers and students and made concrete suggestions about how to perform experiments in order to test his novel hypothesis. He also initiated personal exchange activities and collaborations between the Chinese and oversea scientists to move forward the purine stories in AP.

**Fig. 3:**
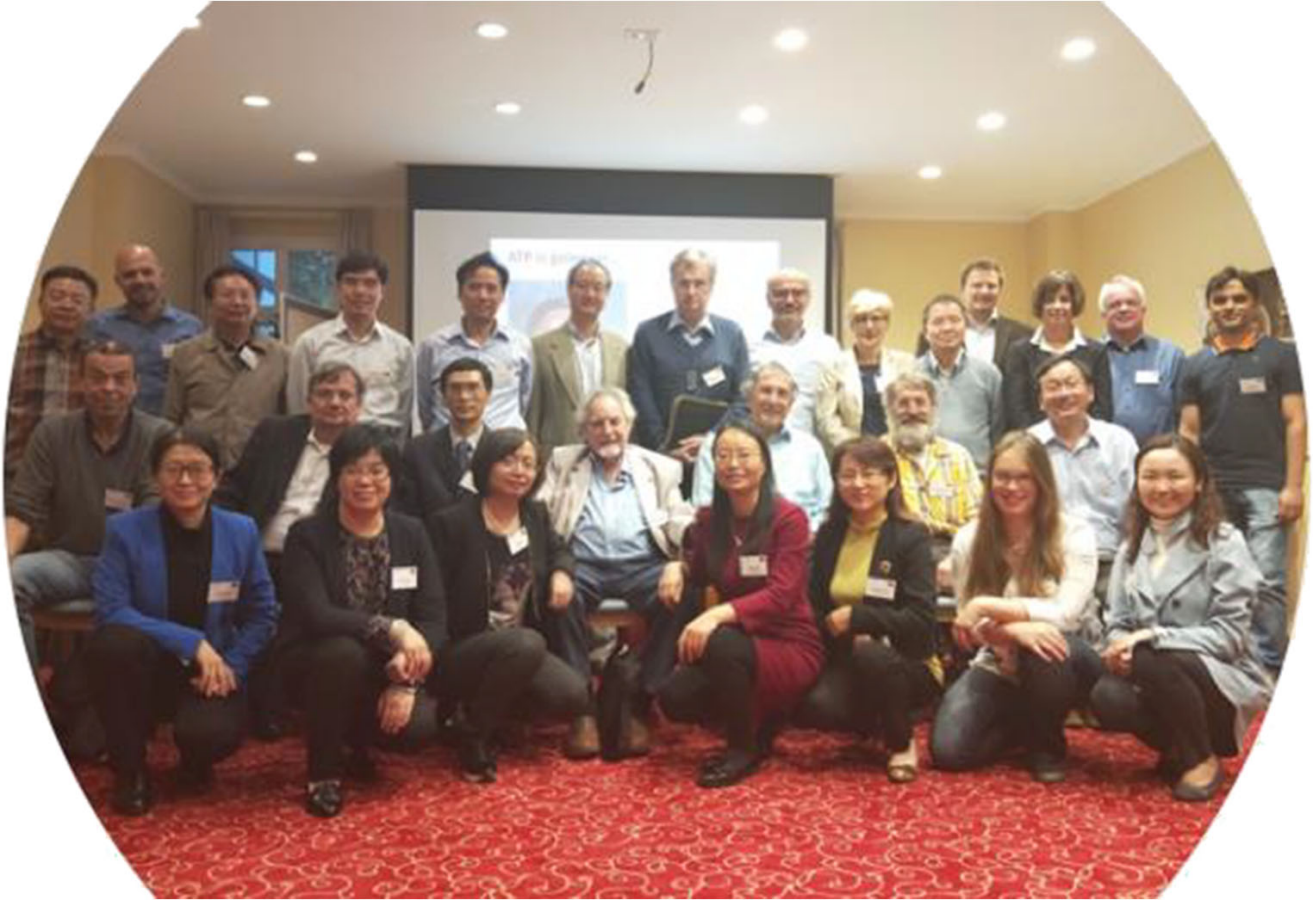
Geoffrey Burnstock, Peter Illes, and Yong Tang at the 2nd Sino-German Symposium on Purinergic Signalling, Pain and Acupuncture in Leipzig, 2017

In 2017, the “2nd Sino-German Symposium on Purinergic Signalling, Pain and Acupuncture” was held in Leipzig. Geoff, at the age of 88, came to Leipzig with his wife Nomi and again gave the opening talk on purinergic signalling and AP. His lecture greatly encouraged the participating scientists (Fig. 3) to pursue the story of purines in AP. Lectures presented by Chinese participants also inspired the prevailing confidence that purinergic signalling will explain the beneficial effects of AP in pain and inflammation, as well as in immune, vascular, respiratory, gastrointestinal, neurological, and psychological disorders.

**Fig. 4:**
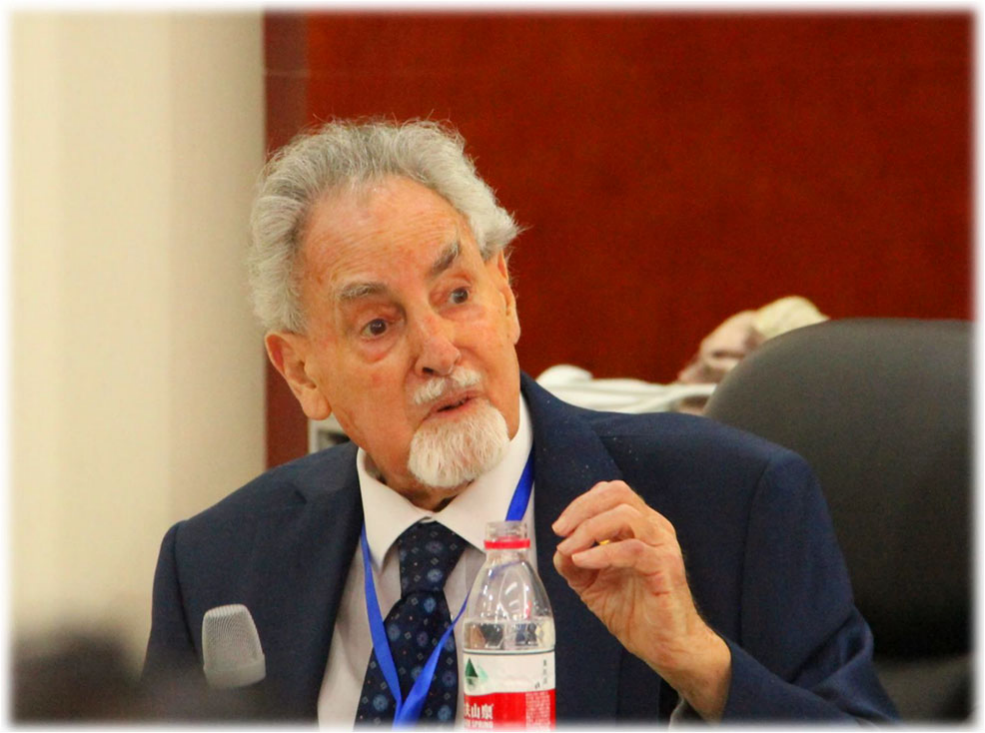
The opening lecture addressed by Geoffrey Burnstock at the 1st Chinese Purine Meeting in Chengdu, 2018

In 2018, Geoff at an age of 89, delivered again a stimulating speech, also as an opening lecture at the “1st Chinese Purine Meeting” when the China Purine Club was founded (Fig. 4). His 1-h presentation let us witness the indomitable energy of the creator of purinergic signalling, even at this advanced age.

**Fig. 5:**
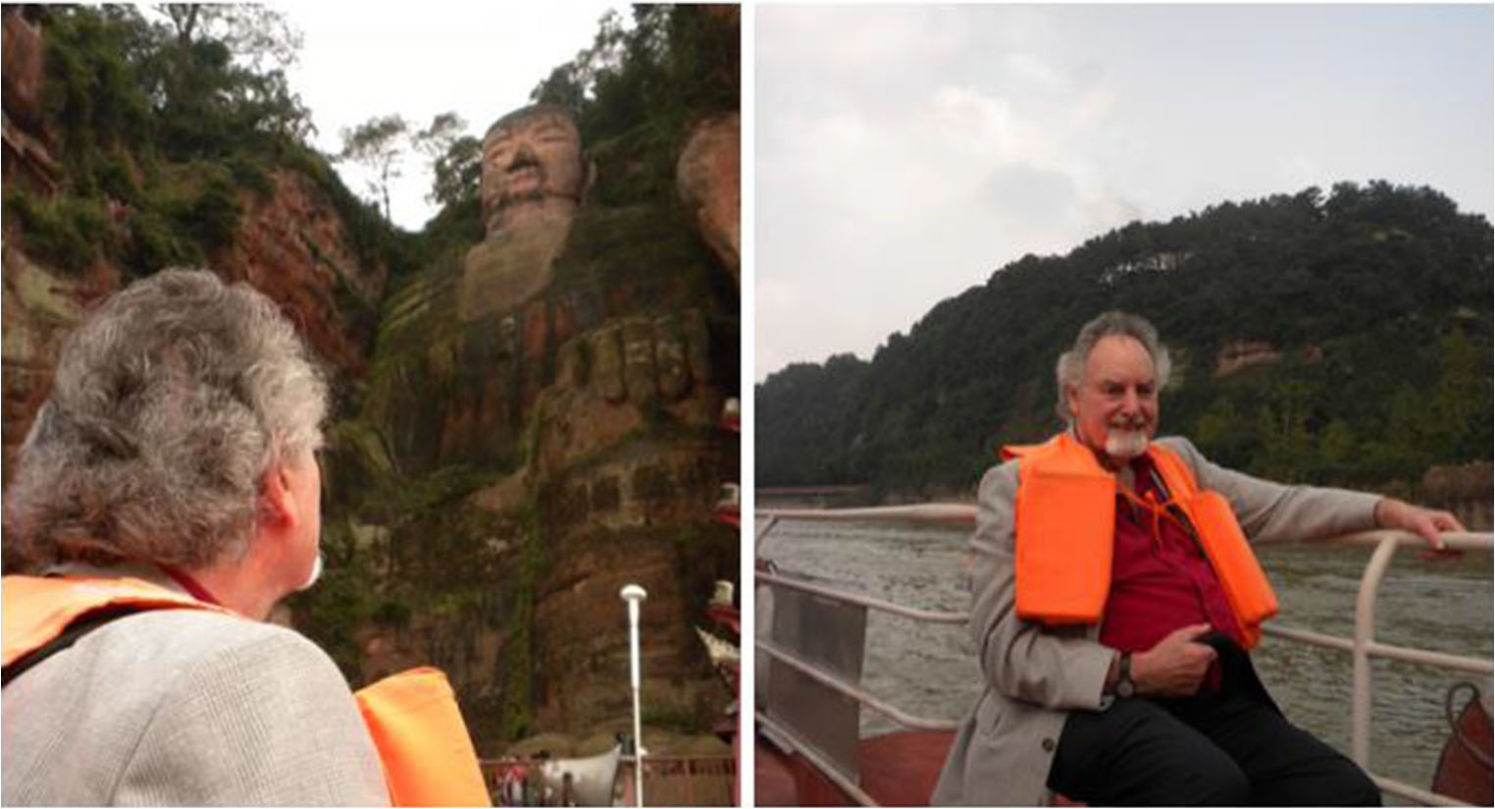
Geoffrey Burnstock visited Leshan Giant Buddha in 2012

The purine story in acupuncture has experienced an astonishing surge in the last two centuries, from zero at the beginning, to up to over 50 publications presently, and it is still in sustained increase. Prof. Geoffrey Burnstock, the father of purinergic signalling, was appointed as Honorary Professor in the Chengdu University of TCM in 2016 and as Honorary President of the China Purine Club in 2018. During the two meetings in China attended by Geoff and Nomi, they visited the old city center of Chengdu, where Geoff enjoyed local sweets, and also visited the southern Yunnan Province of China, the Chendgu Panda base, and Du Fu Cottage, as well as the nearby Leshan Giant Budda (Fig. 5).

Geoff, we will miss you badly in the future.

## My encounters with Geoff (Peter Illes, Leipzig, Germany)

When looking back on my encounters with Geoff, I remember seeing him the first time in 1976 in Varna, Bulgaria, at a Conference organized on Smooth Muscle Physiology. He spoke of course about purinergic transmission in the taenia coli, eloquent as always, and I was most impressed by his talk. At this time, I was a resident of my hometown, Budapest (Hungary), where I had been working at the Department of Pharmacology of the Semmelweis University of Medicine for 10 years after graduation from Medical School. I was deeply impressed by Geoff’s talk and was able to present to him my poster on sympathetic transmission in the guinea-pig vas deferens, which he criticized in a friendly, but decisive manner, and in the retrospect, quite correctly.

The second time I came into contact with him, I was working in Freiburg (Germany) where I stayed from 1981 onwards, at the Department of Pharmacology of the University, as a naturalized German citizen. In 1983, I published a review on noradrenaline as a neuroeffector transmitter in the vas deferens, and discussed at length the riddle of why α- and β-adrenoceptor antagonists fail to block the excitatory junction potentials known to induce mechanical contractions in this organ [58]. The current idea was that although noradrenaline is released from the sympathetic nerve terminals and acts at postsynaptic α-adrenoceptors located at the smooth muscle, a basement membrane generates an anatomical barrier to block the free diffusion of the abovementioned antagonists to the receptor. Another hypothesis even suggested that a third type of adrenoceptor termed “γ” mediated the noradrenaline effects. Geoff sent me a letter in which he called my attention to the purinergic hypothesis and the strong evidence that the co-transmitter ATP rather than noradrenaline itself is the neuroeffector transmitter. Soon afterwards, I became also an enthusiastic advocate of this idea and joined the purinergic community.

Subsequently, he furthered my career many times, for example by inviting me to join the Editorial Board of the newly founded journal *Purinergic Signalling*, or supporting the Leipzig application for a Dislocated Research Group in 2007. It was a difficult task to convince the responsible bureaucrats of the German Research Council that such an initiative made sense. Geoff was a member of the Board evaluating our application and enthusiastically supported it. In consequence, the Research Group became established and was very productive over its 6-year period of functioning [59].

I had the opportunity to meet Geoff at many International Purine Congresses and scientific events in diverse countries. Especially memorable were our encounters in Budapest on occasion of him becoming Honorary Member of the Hungarian Pharmacological Society in 2006, and in Leipzig, on occasion of the awarding of an Honorary Doctorate of our University (2011). In both cases, he was accompanied by Nomi and we spent many hours enjoying animated conversation, good food, and tasty wine.

I have co-authored nine publications with Geoff, both original reports and review articles. One of them was especially successful, becoming a “highly cited paper” according to ISI Web of Science [60]. During our accidental co-operation, I learned to admire his sharp intellect and gift to describe complicated scientific processes in understandable and concise wording.

He had probably the greatest influence on my “late” scientific career after having retired as a Chairman of the Department of Pharmacology in Leipzig, by establishing a contact with Prof. Yong Tang (Chengdu, China) [57]. Yong has given an account of our common activities in the “Important ideas to promote our knowledge on the natural scientific basis of acupuncture: Yong Tang (Chengdu, China)” section.

In conclusion, let me state that having lost Geoff, as a benevolent friend, fatherly figure, and illuminating ideal is a huge personal hardship.
